# Between Knowledge and Strategy: A Cross-Sectional Study on Financial Literacy, Coping, Consumer Loans, and Payment Preferences Among Young Adults in Sweden

**DOI:** 10.3390/bs15111522

**Published:** 2025-11-08

**Authors:** Henrik Levinsson, Lydia Santérus, Emma Samuelsson

**Affiliations:** Department of Psychology, Lund University, 22100 Lund, Sweden

**Keywords:** financial literacy, coping, consumer financial behaviour, gender

## Abstract

Over-indebtedness is at an all-time high in Sweden. The purpose of this study was to examine the relationships between financial literacy, coping strategies, and consumer financial behaviour. A further aim was to examine gender differences in this context. The study employed a cross-sectional survey design, in which participants (*n* = 2057) responded to questions related to the study variables. The results of the analysis found a significant relationship between financial literacy, problem-focused coping, and direct payment. Alongside this, the results revealed a significant relationship between financial illiteracy, avoidant coping, having consumer loans, and deferred payment strategies. Men were more likely to have consumer loans and to choose credit payment. Women were more likely to choose the Buy-Now-Pay-Later option in comparison to men. The findings of the present study can provide valuable insights on the psychological aspects of consumer financial behaviour. Additionally, it is recommended that financial literacy be continuously investigated as a potential “protective shield,” particularly in relation to psychological well-being, as well as in the context of the need for extended financial education.

## 1. Introduction

Financial debt and the ways in which individuals cope with it is a topic of increasing societal concern, particularly among young adults. In 2021, the television show ‘Squid Game’ premiered on Netflix. The show depicts participants in South Korea competing in a life-or-death game to escape financial debt, highlighting dramatised extremes of financial stress. While fictional, the global fascination with the show reflects the very real psychological impact of over-indebtedness. The global issue of over-indebtedness, alongside widespread fascination with a television show depicting how people cope with financial hardships, raises the question of how these phenomena might relate. While ‘Squid Game’ becomes the most popular show on Netflix, over-indebtedness is at an all-time high in Sweden. According to statistics by the Swedish Enforcement Authority (2025), the total household debt in the country is on the rise, with a rise of 36 percent in two years and 19 million SEK in a year. This development is alarming and reflects the financial hardships of the previous years with inflation and increased interest rates, according to the chief enforcement director Fredrik Rosengren (Swedish Enforcement Authority, 2025). Though being an alarming issue in Sweden, the problem is not isolated, and household debt has risen markedly in most countries that are a part of The Organisation for Economic Co-operation and Development (OECD) (André, 2025). 

The present study investigates how the level of financial literacy and the use of coping strategies relate to whether young adults have consumer loans, as well as to their online payment preferences, with gender included as a control variable. While this study is exploratory, these variables are relevant for understanding patterns in how individuals choose to pay for online purchases, such as preferring direct payment or using short-term credit options like Buy now, pay later (BNPL). Concerning consumer loans, we were particularly interested in exploring whether the respondents had taken out consumer loans more than 1.5 years ago (or 18 months), as prolonged debt may indicate persistent financial difficulties. Previous research has also found associations between maladaptive coping strategies and financial problems, highlighting the relevance of including coping in our analyses. Given that financial literacy is generally lower among women, we also control for gender in our analyses.

BNPL has become increasingly popular and widespread, particularly among young adults and in countries with strong e-commerce, high inflation, and less-strict regulation. Globally, Sweden is among the countries with the highest prevalence of BNPL. At the same time, using BNPL options without fully understanding them can lead to over-indebtedness (Cornelli et al., 2023), which could be considered a public health issue. Becoming over-indebted early in life may have long-lasting negative consequences for the individual. 

The Swedish Enforcement Authority has reported that the total debt amount among young adults has increased, with consumer loans and payday loans representing a significant share. While men account for the majority of debts registered with the Enforcement Authority, a sharp increase among women has been reported, particularly due to online shopping.

Sweden performs well in terms of financial literacy in an international context. Nevertheless, young adults aged 18–29 demonstrate lower financial literacy compared with adults aged 30–59. Within Sweden, this age-related gap is relatively large. From an international perspective, the gap is smaller in many other countries (Swedish Financial Supervisory Authority, 2023). Previous research has also found significant gender differences in financial literacy, with women generally exhibiting lower levels of financial literacy and greater uncertainty.

The studied variables are discussed in the following background section, where their relevance is presented based on previous research and identified knowledge gaps. It is important to note that the topic under study has not been well covered in previous academic literature, particularly with regard to young adults and how financial literacy and psychological factors, such as coping strategies, might influence and relate to individuals’ payment preferences. This underscores the importance of further research on financial and psychological well-being among young adults.

## 2. Financial Literacy

Financial literacy is not only a vital skill for the ability to make healthy financial decisions; it is also categorised as an essential life skill (OECD & OCDE, 2020). Being financially literate and resilient is an essential life skill for managing complex modern financial technologies, inflation, and increased risks of war and climate change (Lusardi & Messy, 2023). In recent years, this became especially evident during the 2020 COVID-19 pandemic, when effective money management and financial cushions played a crucial role in mitigating economic strain and ensuring financial well-being (Lusardi & Mitchell, 2023).

The Organisation for Economic Co-operation and Development (OECD, 2013) defines financial literacy as

The knowledge and understanding of financial concepts and risks, but also the skills, motivation, and confidence to apply such knowledge and understanding in order to make effective decisions across a range of financial contexts, to improve the financial well-being of individuals and society, and to enable participation in economic life (p. 144).

The emergence of easily accessible financial technology (like payday loans) has increased the necessity of financial literacy, as this has put responsibility on the consumer in a way that it has not before (Pillai et al., 2023). Despite its importance, global financial literacy is surprisingly low, even in financially developed countries. In 2015, the percentage of financially literate adults worldwide was 33 percent, meaning that 3.5 billion adults across financially developed countries were unable to make informed economic decisions (Klapper et al., 2015).

There is a clear social disparity in financial literacy, where women and low-income individuals indicate lower financial literacy than men and high-income individuals (Klapper et al., 2015). Furthermore, there is a positive correlation between higher education and higher financial literacy (Demertzis et al., 2024). Also mentioned in the article by Demertzis et al. is that the reason for the social disparity is multi-fold, as it can be due to wage gaps and career interruptions, as well as the fact that not earning as much means not participating in economic life in the same way.

A study investigating gender differences in financial risk-taking found that while gender is not the sole contributor to the difference between men and women, as it is also influenced by income uncertainty and wealth, men are more prone to financial risk-taking (Fisher & Yao, 2017). Lusardi and Mitchell (2014) highlight the global gender difference in financial literacy, regardless of education level and marital status. Single women who oversee their own finances still indicate lower financial literacy than men. Their results underline the need for more research on this topic, as the reason for this is still unclear (Lusardi & Mitchell, 2014). More recent research found a lack of financial confidence in women to be a possible factor for this disparity (Bucher-Koenen et al., 2024). Furthermore, a recent study by Samuelsson et al. (2024) investigating financial literacy, personal financial situation, and mental health in Sweden found that the financial literacy amongst young adults is lower than anticipated, and that women indicate lower financial literacy.

### 2.1. Financial Technology (FinTech)

The digital shift in how finances are handled may have had an impact on financial well-being and global financial illiteracy (Panos & Wilson, 2020). Financial technology, or FinTech, is a relatively new phenomenon that encapsulates the redefinition of the financial sector in the digital age. Gomber et al. (2017) defines FinTech as “a neologism which originates from the words “financial” and “technology” and describes in general the connection of modern and mainly, Internet-related technologies (e.g., cloud computing, mobile Internet) with established business activities of the financial services industry (e.g., money lending, transaction banking)” (p. 540). A study investigating the relationship between household indebtedness and the increased use of FinTech found that FinTech development intensifies the probability of household debt expansion, resulting in financial crisis (Yuan et al., 2024). Hundtofte and Gladstone (2017) imply a correlation between phone usage and impulsive consumer behaviour, and the increased likelihood of applying for payday loans. Additionally, a study conducted in Poland comparing young adults and adults in relation to attitudes against using FinTech products and services found that the younger generation was significantly more likely to use them (Krupa & Buszko, 2023).

### 2.2. Financial Literacy and Debt

Financial literacy might have an impact on the tendency of debt. Tang (2022) investigated the relationship between financial literacy and consumer debt in China and found that high financial literacy is linked to household debt, while low financial literacy is linked to excessive debt. Although having household debt, such as mortgages and student loans, is common and often necessary, individuals with higher financial literacy may be better equipped to avoid over-indebtedness. A study investigating financial literacy and impulsivity as predictors of debt burden found that people with low financial literacy tend to have higher debt, but this is also due to them being more impulsive (Ottaviani & Vandone, 2017). A survey study from 2021 found that people with financial literacy are less affected by over-indebtedness than people without (Kurowski, 2021).

## 3. Consumer Financial Behaviour

In the present study, “Consumer Financial Behaviour” refers to individuals’ preferences for financing their purchases and investments. The financing strategies examined include consumer loans, the Buy-Now-Pay-Later-Model (BNPL), credit payment, partial payment, and direct payment.

### 3.1. Consumer Loans

Consumer loans refer to financing for personal or household purposes, such as mortgage loans, credit card, and student loans (Federal Deposit Insurance Corporation (FDIC), 2024). A report by the Swedish Financial Supervisory Authority (2020b) states that the amount of personal loans and credit-based borrowing is increasing, with the elevated access of digital lending being a possible factor of this progression, such as the BNPL model. It also accounts for the age difference in consumer lending, where younger people tend to take out smaller loans in comparison to older individuals, who take out loans for housing or debt consolidation to a greater extent.

The increased consumer debt entails risks on both individual households and lenders. An academic research article discussing consumer loans in a European context problematizes the unregulated access to consumer loans and highlights the increased risk of over-indebtedness and financial instability on both a personal and systematic level (Ferretti, 2010). Late loan payments and loan defaults have risen according to the report by Swedish Financial Supervisory Authority (2020a) (hereafter referred to as SFSA), and over-indebtedness is a growing concern, especially among younger and low-income individuals due to poor financial planning and knowledge, as well as situational factors such as loss of employment. The report also problematizes the increased debt accumulation (taking out loans to cover already existing ones) leading to a vicious cycle of a never-ending indebtedness.

### 3.2. Buy-Now-Pay-Later Model (BNPL)

The Buy-Now-Pay-Later model, or BNPL, is an interest-free credit option for retail purchases that typically entails a 30-day invoice. This payment method has become increasingly popular through payment platforms such as Klarna and Afterpay (Federal Reserve Bank of Boston, 2024). It is typically used for online purchases on e-commerce platforms but is becoming increasingly popular in stores with scannable barcodes as well. The use of BNPL has increased more than six-fold between 2019 and 2023, with a noticeable change during the COVID-19 pandemic. Based on a survey from the United States, BNPL is used among the younger generation and among individuals with lower incomes and lower educational levels to a greater extent (Cornelli et al., 2023). Furthermore, a study by Waliszewski et al. (2024) investigating how consumer behaviour and sociodemographic aspects influence the use of BNPL found significant differences based on income, gender, and education, as well as innovativeness, family history of loans, and inclination of going into debt. Ackert et al. (2024) found that people are more inclined to use the BNPL method in comparison to credit card loans, due to societal norms and it being perceived as a good choice.

One explanation for the BNPL phenomenon can be found in behavioural economics, particularly the concept of hyperbolic discounting, which refers to the tendency of individuals to make decisions that are based on immediate rewards rather than delayed rewards (Enke et al., 2023). This intertemporal behaviour explains why people discount future payment and take on credit card debt despite the high interest rates, for example (Mallard, 2017). A study from 2024 investigated the relationship between financial literacy and the hyperbolic discounting bias and found that financial knowledge, behaviour, and attitude have significant impacts on the intertemporal preferences in financial decision-making, thus highlighting the importance of these aspects on the reduction in hyperbolic economic decision-making (Bawalle et al., 2024).

### 3.3. Credit and Partial Payment

With the increase in e-commerce, more people are using credit payment. Credit payment refers to purchasing something and paying it off at a later date, which usually occurs by purchase through a credit card. Credit payment is not necessarily negative, but it increases the risk of purchasing over one’s means and comes with additional cost in the form of interest and processing fees, for example (Swedish Consumer Agency, 2023a). Aydin (2021) investigated psychological and demographic factors influencing responsible credit card debt payment and found that more optimistic, intuitive, and materialistic individuals were more responsible with their credit card payments. Partial payment is a type of credit payment where the payment is made by several, smaller payments over time instead of the entire amount at once (Swedbank Pay, 2025). This payment method is typically not interest-free.

Importantly, a report by the Swedish Consumer Agency (2023b) found that 38 percent of people do not perceive their credit card use as making purchases on credit, even when the charges are accumulated over a period and later paid in instalments. This could be due to how easily accessible these payment options have become.

## 4. Indebtedness and Mental Health

There is a clear correlation between being in debt and poor mental health. Indebtedness is common in and associated with higher levels of depression and anxiety (Drentea & Reynolds, 2012). A systematic review found several studies indicating a relationship between financial hardships and poor mental health. A cohort study conducted in the United States found that drops in household incomes were positively correlated with anxiety, depression, and substance-use disorders (Sareen et al., 2011). Furthermore, a longitudinal study found that household payment problems and indebtedness are detrimental to mental health (Taylor et al., 2007). To handle this financial strain, one might turn to various coping strategies. A study from 2024 examined the effects of coping strategies on mental health during the COVID-19 pandemic and found that problem-focused coping was associated with lower odds of depression, while avoidant coping increased the likelihood of both anxiety and depression (Chang et al., 2024).

### Over-Indebtedness

Over-indebtedness is a complex term, and there is not one accepted definition of over-indebtedness as it can entail various factors. However, Östergren et al. (2022) coined the overall definition of “a situation, persisting for a sustained period of time, in which daily cost of living exceeds income, and in which the imbalance cannot be compensated by savings” (p. 2). An over-indebted population is an issue on multiple levels. On a subjective note, an excessive amount of debt might affect social and familial relations, and lead to social and psychological distress, social embarrassment, internalised shame, and suicidal behaviour (Bialowolski et al., 2024; Andersson & Pozo Pérez, 2024; Levinsson et al., 2023; Rojas, 2022), making the area of over-indebtedness important for managing psychological and social well-being. Based on recent reports from Sweden, there is an increasing issue with young women who are turning to prostitution to pay off their debts, or to pay rent and to afford food (Radio Sweden, 2025). On a larger scale, an over-indebted population affects economic participation and national economic stability by decreasing the gross domestic product (GDP) and increasing the risk of banking crises (Alter et al., 2018). There can be several culprits behind increased household debts. In an economic analysis report by SFSA, household debt has increased due to a combination between rising household costs, macroeconomic factors, and consumption behaviour (Bäckman et al., 2021).

## 5. Coping Behaviours

Coping behaviours refer to the act of soothing oneself after a stressful situation (Henson et al., 2012). In 1984, Lazarus and Folkman laid the foundation to the ‘Coping Theory’, which distinguished two primary coping strategies: problem-focused and emotion-focused. They define coping as “constantly changing cognitive and behavioural efforts to manage specific external and/or internal demands that are appraised as taxing or exceeding the resources of the person” (Lazarus & Folkman, 1984, p. 141). In research, coping strategies are commonly categorised into three main types: problem-focused coping, emotion-focused coping, and maladaptive coping, although alternative categorisations also exist.

Problem-focused coping refers to the act of identifying a problem and actively trying to solve it without being overwhelmed by emotions (Lazarus & Folkman, 1984). A study investigating the effect of problem-focused coping on emotional clarity and mental health among older adults found that dealing with issues in a problem-focused manner indicated higher life satisfaction and lower depression levels (Cho & Choi, 2024). Additionally, a study investigating psychological capital (the ability to manage tough situations) and problem-focused coping found a positive predicting effect between the two, meaning people who use problem-focused coping are better equipped to handle adverse conditions (Wang et al., 2022). Emotion-focused coping refers to the act of regulating the emotional response to the stressful situation by using cognitive and behavioural tools to reduce the emotional response, such as emotional support or distractions (Lazarus & Folkman, 1984). Maladaptive coping refers to dealing with negative emotions in a destructive way, such as avoidance, self-blame, or compulsive behaviours (Wadsworth, 2015). Compulsive behaviours can entail substance abuse, eating disorders, and excessive buying (e.g., retail therapy) (Joseph et al., 2022), which might all lead to long-term consequences such as poor life quality, declining health, and over-indebtedness.

### Financial Situation, Mental Health, and Coping Behaviour

The way in which indebted individuals and households cope with their financial strain is based on several different factors. A study by Tach and Greene (2014) explains this in two broad categories: assistance, where one turns to external sources for help, such as family and friends, and which could be categorised as emotion-focused coping, and individualised strategies, such as debt juggling and taking on extra jobs, which could be categorised as problem-focused coping. The individualised approach is more common, as this maintains the self-sufficient identity, and asking for assistance is seen as the absolute last resort. In 2019, Holmgren et al. explored coping strategies amongst over-indebted individuals regarding mental illness and found that those who were over-indebted and indicated mental health issues were more likely to use maladaptive coping strategies. Furthermore, a study investigating financial stress and coping amongst college students found that those who were experiencing financial stress were more likely to engage in risky coping behaviours (Britt et al., 2016). While debt might increase mental health issues such as depression and anxiety, financial knowledge and self-efficacy can buffer these effects (Kim & Chatterjee, 2021). This is also investigated in a study by Serido et al. (2010), who found that psychological well-being was positively correlated with proactive and preventative financial coping, such as budgeting and saving.

## 6. Aims and Research Questions

Previous research explains a lack of financial literacy, not only in Sweden, but also globally. It highlights why financial literacy is crucial and how low financial literacy might lead to problematic financial behaviours. Even though there is research on financial coping, there is limited research on how financial literacy and psychological factors such as coping strategies might influence and relate to personal payment preferences. Additionally, the specific age group of young adults (18–29) is not a particularly researched target group within this field.

The aim of the present study is to obtain a deeper understanding of how financial literacy and coping behaviours relate to whether one has consumer loans and personal payment preferences. It also seeks to explore gender differences by examining how men and women vary in this context. 

The significance of this research is due to the rising and increasing issue of over-indebtedness in Sweden, especially among the younger generation, and the damaging psychological consequences of being over-indebted. It might provide valuable insights into how financial literacy and coping behaviours relate to financial behaviour, and how this might differ between young men and women. The purpose of the present study will be to determine the answers to the following research questions:

(1) Is there a relationship between (a) having consumer loans and financial literacy, (b) having consumer loans and coping strategies (problem-focused, emotion-focused, and avoidant), and (c) financial literacy and coping strategies?
(2a) Can financial literacy and coping strategies predict having consumer loans?
(2b) Can financial literacy and coping strategies predict personal payment preferences?
(3) If these relationships exist, do they remain when controlling for gender?

## 7. Materials and Methods

The following section describes the study procedure, the participants, the measurements used, the data analysis, and ethical considerations.

### 7.1. Design, Procedure, and Participants

The study is a cross-sectional study with a quantitative approach. The quantitative method was applied based on the study’s aim of finding relationships and predictors of the included variables based on a larger sample size. The survey consisted of questions measuring financial literacy, personal financial situation, coping, loneliness, sense of belonging, and mental health.

The survey was open for participation for two weeks in October of 2021. Data was collected via the Swedish research company Enkätfabriken (https://www.enkatfabriken.se/, accessed on 15 May 2025). Enkätfabriken is one of Sweden’s biggest survey companies and recruit respondents who have agreed to participate in surveys via a web panel. They aim to recruit a representative sample through probability sampling and to recruit a diverse sample regarding gender, age, and geographic location. The inclusion criteria for participating in the survey was that the respondent should be 18 years old or above, be able to read and understand Swedish, and have access to an internet connection. Upon completion of the data collection, a total of 2057 people answered the survey. The dataset consists of women and men aged 18–29 from all around Sweden. The full demographic information can be found in Table 1.

### 7.2. Materials

#### 7.2.1. Financial Literacy

To measure financial literacy, the respondents were asked to answer three questions regarding their financial knowledge with the ‘Big Three’ measure. The ‘Big Three’ measure was created by Lusardi and Mitchell (2011) and measures the respondent’s financial literacy through four key principles: simplicity, relevance, brevity, and capacity to differentiate. When designing the measure, they identified three basic economic principles that people should have some understanding of; these are (i) understanding of interest compounding; (ii) understanding of inflation, and (iii) understanding of risk-diversification (Lusardi & Mitchell, 2011). Included in the ‘Big Three’ measure are the questions:

1. Suppose you had SEK 100 in a savings account and the interest rate was 2% per year. After 5 years, how much do you think you would have in the account if you left the money to grow?
2. Imagine that the interest rate on your savings account was 1% per year and inflation was 2% per year. After 1 year, how much would you be able to buy with the money in this account?
3. Please tell me whether this statement is true or false. ‘Buying a single company’s stock usually provides a safer return than a stock mutual fund’.

All questions are multiple-choice, with only one correct answer. Each question always includes the option “Do not know”, which is considered as a lack of financial literacy. A high score on the ‘Big Three’ questions indicates savvy financial behaviour, in for instance, retirement planning and debt management, and could therefore be indicative of financial well-being. The respondents who answered all three questions correctly were categorised as “financially literate” while all remaining respondents as “not financially literate”. The scale has been implemented in several surveys, such as the National Longitudinal Survey of Youth (NLSY), the National Financial Capability Study (NFCS), and the survey of Consumer Finances (SCF), to name a few (Lusardi & Mitchell, 2014). In the current study, the reliability of the ‘Big Three’ scale only yielded Cronbach’s α = 0.51, but this can be explained due to there only being three items. However, the average inter-item correlation between items was 0.26, which is within the recommended span of 0.2 and 0.4 (Piedmont, 2014). The Swedish version of the ‘Big Three’ questions was taken from a report by the Swedish Financial Supervisory Authority (2020a).

#### 7.2.2. Coping

To measure the respondents’ coping behaviour, they were asked to answer the BriefCOPE inventory. The BriefCOPE was developed in 1997 as a shortened version of the 60-item ‘Coping Orientation to Problems Experienced Inventory’ by Carver et al. (1989) and instead features 28 items measuring the coping style of the respondent (Carver, 1997). In 2012, Dias et al. (2012) conducted a psychometric analysis of the measure and divided it into three subscales: problem-focused coping, emotion-focused coping, and avoidant coping. This categorisation has been used in the present study. From a reliability analysis in the present study, the Cronbach’s α for the coping behaviour measures were α = 0.80 for problem-focused, α = 0.82 for avoidant, and α = 0.68 for emotion-focused, meaning that the emotion-focused measure did not meet the standard for reliability with Cronbach’s α (>0.70). The three-level categorisation has also been validated in a study by Poulus et al. (2020), which found the reliability satisfactory with Cronbach’s α at 0.81 for the problem-focused and 0.75 for the emotion-focused categories. The avoidant category only reached 0.68, meaning it did not meet the standard. However, based on a confirmatory factor analysis (CFA), the results indicate a strong overall model fit, and the measure is deemed applicable (Poulus et al., 2020). The measure includes questions such as “I’ve been trying to come up with a strategy about what to do” (problem-focused), “I’ve been getting emotional support from others” (emotion-focused), and “I’ve been using alcohol or drugs to help me get through it” (avoidant). The measure is a 4-point Likert scale ranging between “I haven’t been doing this at all” to “I’ve been doing this a lot”. Interpretation of the results is performed by the sum of each question in respective three-level categorisation. For example, the sum of all questions regarding avoidant coping = level of avoidant coping. The maximum number of points to be scored for the problem-focused and avoidant categorizations is 32 points and 48 for the emotion-focused categorisation. The Swedish translation of the instrument was made by Muhonen and Torkelson (2001).

### 7.3. Consumer Financial Behaviour

To measure the respondents’ consumer financial behaviour, they were asked about consumer loans and their personal payment preferences. The question regarding consumer loans asked whether they have a consumer loan that was taken out more than 18 months ago. This has the aim of targeting the respondents with long-term debt, as this could be indicative of financial difficulties. The question on personal payment preference examined how participants would most likely pay for an online purchase and was divided into the most common alternatives with the following response options: “Direct”, “30-day invoice”, “Credit”, and “Partial payment”. The respondents who answered “30-day invoice” were categorised as users of the Buy-Now-Pay-Later model (BNPL).

## 8. Data Analysis

The data was extracted and analysed in ‘Jamovi’ (version 2.6.44). Some data was re-coded to be able to conduct adequate analyses, such as the ‘Big Three’ financial literacy questions and the BriefCOPE scale. The significance level was set to *p* < 0.05. To assess normality, skewness and kurtosis was examined, providing satisfactory results. The Shapiro–Wilk test indicated deviations from normality, but due to the large sample size, (n > 2000) the effect is minimal on the results.

The correlational analysis between financial literacy, coping strategies, and consumer financial behaviour was carried out using Pearson’s correlation coefficient (*r*) and Phi (Φ) for association between two categorical variables. Importantly, payment preferences were excluded from the correlation matrix due to the risk of uninterpretable results from categorical variables. Binomial and multinomial hierarchical logistic regressions were applied to find the effects of coping strategies, financial literacy, and gender on the odds of engaging in specific financial behaviours (consumer loans and payment preference). The predictors in the regression models were entered in blocks based on theoretical reasoning and the assumed significance for the outcome. Financial literacy was added first, due to its proven importance in financial behaviour. Coping strategies were added second to examine the potential psychological processes influencing financial behaviour. Gender was added third to assess individual-level differences in financial habits. Model fit was assessed using the Akaike information criterion (AIC). Nagelkerke’s R^2^ was used to assess pseudo-explained variance (proportion of variance). Chi-square (χ^2^) was used to compare model fit to the null model. Odds ratios (ORs) with 95% confidence intervals (CIs) were calculated to assess the strength and precision of the associations between predictors and outcomes in the regression analyses. Variance inflation factors (VIFs) were used to assess multicollinearity to ensure it did not reach levels that would compromise the admissibility of the estimations.

## 9. Ethical Considerations

The study was not subject to ethical review under Swedish law (2003:460) (Swedish Parliament, n.d.), as the survey was anonymous and no personal data were collected. Participants were informed that their participation was voluntary and that they could withdraw at any time without providing a reason. The survey included questions on topics that could, in some cases, lead to distress, such as personal finances, loneliness, depression, and anxiety. Therefore, at the beginning of the survey, participants were informed that if they experienced distress, they could seek help from healthcare services. After reading this introductory information, participants gave informed consent before proceeding to the actual survey.

## 10. Results

In the following section, the results of all statistical investigations will be presented. To answer the study’s research questions, correlations have been tested through a correlation matrix and binomial and multinomial hierarchical logistic regressions.

### 10.1. Descriptive Results

Table 2 shows the descriptive results for all variables by gender.

Question 1: Is there a relationship between (a) having consumer loans and financial literacy, (b) having consumer loans and coping strategies (problem-focused, emotion-focused, and avoidant), and (c) financial literacy and coping strategies? See Table 3 for correlations between the variables.

The correlation matrix indicates significant correlations between some of the variables. A significant association was found between consumer loans and financial literacy. Additionally, consumer loans were correlated with problem-focused coping and with avoidant coping. Financial literacy was also correlated with problem-focused coping and with avoidant coping. The coping strategies are correlated with each other, except for avoidant and problem-focused.

Question 2a and 3: Can financial literacy and coping strategies predict having consumer loans? Do these relationships remain when controlling for gender?

To examine if financial literacy, coping strategies, and gender could predict the odds of having consumer loans, a binomial hierarchical regression analysis was conducted. Results of the final model (Model 3) can be found in Table 4. 

As shown in Table 4, financial literacy turned out to be a significant predictor, and financially literate individuals were less likely to have consumer loans. For the coping strategies, problem-focused coping was a significant predictor, where individuals who engage in more problem-focused coping were less likely to have consumer loans. In contrast, avoidant coping was negatively significant, indicating that more avoidant individuals were more likely to have consumer loans. Emotion-focused coping was not significant. In this sample, men are more likely to have consumer loans in comparison to women.

Question 2b and 3: Can financial literacy and coping strategies predict personal payment preference? Do these relationships remain when controlling for gender?

To examine if financial literacy, coping strategies, and gender could predict payment preference (BNPL, credit, partial in comparison to direct), a multinomial hierarchical regression analysis was conducted. Payment preferences were excluded from the correlation matrix (see Table 3) due to the risk of uninterpretable results from categorical variables. The results of significant predictors in the final model (Model 3) can be found in Table 5.

### 10.2. Payment Preference

The multinomial regression analysis indicated some significant results with regard to questions 2b and 3. With regard to the usage of BNPL, the results indicate that men are less likely to use BNPL in comparison to women. Neither coping strategy or financial literacy significantly predicted the use of BNPL. Some of the variables significantly predicted the usage of credit payment. Problem-focused coping was negatively associated with credit payment, indicating that individuals who cope in this manner are less likely to use credit payment. In contrast, avoidant coping was positively associated with credit payment, indicating higher odds of using credit payment. Emotion-focused coping was not significant. Men are more likely to use credit payment in comparison to women. Financial literacy significantly predicted the use of partial payment with a negative association, indicating that individuals without financial literacy are more likely to use partial payments. Two of the coping strategies were significant, with problem-focused coping being negatively associated and avoidant coping being positively associated, indicating that individuals engaging in more problem-focused coping might be less likely to use partial payments while people who engage in avoidant coping might be more likely to use it. Emotion-focused coping was not significant. There was no significant association between gender and the use of partial payment.

### 10.3. Discussion

The present study aimed to investigate relationships between coping strategies (problem-focused, emotion-focused, and avoidant), financial literacy, and consumer financial behaviour in terms of having consumer loans and personal payment preferences. The statistical analysis revealed several significant findings. The following section will reflect on these findings in relation to previous research and knowledge.

#### 10.3.1. Is There a Relationship Between (a) Having Consumer Loans and Financial Literacy, (b) Having Consumer Loans and Coping Strategies (Problem-Focused, Emotion-Focused, and Avoidant), and (c) Financial Literacy and Coping Strategies?

A weak positive association could be found between financial literacy and consumer loans, suggesting a relationship between the two. This significant relation aligns with the report by the Swedish Financial Supervisory Authority (2020b) which stated a correlation between late loan payments and over-indebtedness, possibly due to a lack of financial knowledge. Additionally, this is consistent with earlier research by Tang (2022), Ottaviani and Vandone (2017), and Kurowski (2021), who all found financial literacy to be related to indebtedness. A positive association could be found between consumer loans and problem-focused coping, and a negative association with avoidant coping, pointing to a relationship between financial decisions and coping behaviour. This reflects earlier work which found that financial stress and over-indebtedness was related to more maladaptive coping behaviours (Britt et al., 2016; Holmgren et al., 2019).

The correlational analysis revealed a strong positive association between financial literacy and problem-focused coping and a strong negative association with avoidant coping, further strengthening the suggested relationship between finance and coping behaviours, here in the form of financial knowledge.

Emotion-focused coping was not significantly associated with consumer loans or financial literacy in this sample; therefore, these results do not support the involvement of emotion-focused coping. This could be due to emotion-focused coping being more centred around social comfort rather than individual coping (Lazarus & Folkman, 1984). The previous research by Tach and Greene (2014) stated that the individualised approach is more common in debt-handling because it maintains self-sufficiency.

#### 10.3.2. Can Financial Literacy and Coping Strategy Predict Having Consumer Loans?

To further understand the relationship between financial literacy, coping strategies, and consumer loans, a hierarchical regression analysis was conducted to examine if these variables could predict the usage of consumer loans. In this analysis, having financial literacy was associated with lower odds of having consumer loans. This also aligns with previous research and reports that problematise the usage of consumer loans and the increased risk of over-indebtedness (Ferretti, 2010; Swedish Financial Supervisory Authority, 2020b), and indicates that individuals with low financial literacy might be more prone to taking out consumer loans due to having lower knowledge of the risks. These results are further strengthened by previous research which found that low financial literacy might increase the likelihood of being over-indebted (Tang, 2022; Ottaviani & Vandone, 2017; Kurowski, 2021).

In addition to financial literacy, the analysis suggested that individuals who use problem-focused coping strategies were less likely to have consumer loans, whereas individuals who use avoidant coping strategies were more likely to have consumer loans. This could explain that problem-focused individuals cope with financial matters in a more preventative and proactive way, rather than to take out loans, aligned with the previous research by Serido et al. (2010), who found that psychological well-being was positively correlated with proactive and preventative financial coping. Additionally, based on the previous research by Cho and Choi (2024), who found that problem-focused individuals rated themselves higher on life satisfaction and psychological well-being, this could indicate that problem-focused individuals who display higher psychological well-being make more problem-focused financial decisions (proactive and preventative) and therefore take out less consumer loans. Avoidantly coping individuals are more likely to engage in maladaptive behaviours, such as compulsive buying, and might therefore take out more loans to finance purchases (Joseph et al., 2022).

Emotion-focused coping was not a significant predictor, maintaining the present study’s inability to explain any involvement of this particular coping strategy on financial behaviour.

#### 10.3.3. Can Financial Literacy and Coping Strategy Predict Personal Payment Preference?

Problem-focused coping was a significant negative predictor in credit and partial payment, suggesting that individuals who are more problem-focused use these payment options less. In line with the previous research by Serido et al. (2010), this could also be explained by the psychological well-being associated with problem-focused coping, which in turn could promote more preventative financial decisions. Alongside this, financial literacy was negatively related to credit and partial payment, indicating that financially literate individuals were also less likely to use credit and partial payments. Problem-focused coping and financial literacy were positively related to each other, but they both predicted a lower use of credit and partial payment, and therefore might contribute to more financially responsible behaviour. Aligned with the findings of Kim and Chatterjee (2021), being financially literate and confident in one’s financial abilities may help mitigate the mental health effects of debt. Similarly, problem-focused coping is positively associated with psychological well-being (Cho & Choi, 2024) and direct payments, suggesting an association between all three. All coping strategies and financial literacy were unexpectedly not significant to the use of BNPL, which could be situational and specific to this particular sample, and invites further research on this topic.

When examining these results through the theory of hyperbolic discounting, it is not strange that people favour credit and partial payment over direct payment. Individuals tend to prefer immediate rewards, without fully considering future consequences. Aligned with the study from Bawalle et al. (2024), the result from this study indicates that there is a relationship between financial literacy and choosing direct payment over credit and partial payments. Adding coping mechanisms to the theory, avoidant individuals might use consumerism to relieve psychological distress, and are more likely to finance this with credit and loans in comparison to problem-focused individuals.

#### 10.3.4. Do These Relationships Remain When Controlling for Gender?

The analysis indicated some gender differences in financial literacy and behaviour. Regarding consumer loans, the results suggest that men are more likely to have consumer loans than women. This is in accordance with a report by the Swedish Financial Supervisory Authority (2021) that states that men have larger consumer loans in comparison to women due to a generally larger financial income and increased risk-taking. This is also supported by the previous study highlighting that men tend to take more financial risks (Fisher & Yao, 2017). Furthermore, Meyll and Pauls (2019) found that women are less likely to become over-indebted in comparison to men, and that factors such as sociodemographic status, financial literacy, and risk attitude fail to explain this disparity. This aligns with the present study, where men indicate higher financial literacy, while at the same time having more consumer loans.

Differences in gender could also be found between the different payment options. Men were less likely to use the Buy-Now-Pay-Later model (BNPL) in comparison to women, which can also be supported in the report by the Swedish Financial Supervisory Authority (2021) which states that women have more loans than men as they are more prone to taking out smaller loans such as invoice and partial payment in comparison to credit payment, which is more common amongst men. This is indicated in this study’s sample as well.

## 11. Limitations

Commonly used and validated instruments have been used to examine the relationship between financial literacy and coping strategies on financial consumer behaviour, but there are limitations to consider when drawing conclusions from the results of this study.

The BriefCOPE instrument does not specifically measure financial coping, which might threaten the construct’s validity, but the significant results indicate a relationship between coping and financial decisions and knowledge, allowing for further exploration. The variable targeting consumer loans is self-constructed and was limited to a span of 18 months (1.5 years) in an attempt to address persistent and prolonged financial issues. However, this particular timespan can be subject to further reflection.

The large sample size enables for a representative reflection of this particular Swedish population, as well as to conduct statistical investigations of the data. However, it is important to take into consideration that a survey research method might threaten ecological validity due to possible behavioural differences and response biases in the respondents. This is especially relevant in the context of personal finances and coping, which could be topics where individuals may want to present themselves in a socially desirable way.

The participants in this study were collected through a web panel specifically targeting individuals who have accepted survey participation. With this in mind, one should be cautious about representativity; young adults most affected by financial difficulties are less likely to participate in surveys addressing these issues. The data being collected through a web panel and from individuals who have specifically accepted participating in surveys might threaten the external validity due to selection bias. However, the complete anonymity of the survey minimises the potential response bias in the sample. Future research would benefit from examining public registers on debts and mental health indicators, thereby reducing self-reporting bias.

Although statistically significant, as indicated by the stable Wald statistics (e.g., financial literacy, avoidant coping, gender), the overall models explained only a small portion of variance in outcomes. This is something to take into consideration. However, while several factors contribute to explaining consumer financial behaviour, coping is an important psychological piece of the puzzle that is often overlooked. Furthermore, the interpretation of the ‘Big Three’ financial literacy questions was discussed during the statistical investigations in this study, and whether the data should be continuously or nominally interpreted. In our conclusion, the division ended up entailing the participants who answered all three questions correctly as financially literate, and the rest as not. It might be worth exploring the ‘Big Three’ variable as continuous, and with respondents scoring in the categories of ‘high’, ‘medium’, and ‘low’ financial literacy instead of ‘yes’ or ‘no’, for the possibility of more nuanced information.

The present study is cross-sectional; therefore, it cannot claim any causal relationships. However, this was not the aim of the study, which was to find potential associations between financial literacy, coping strategies, and consumer financial behaviour. Therefore, even though the study has found correlations between the included variables, causal relationships should not be assumed. Rather, the findings can be considered hypothesis-generating and serve as a basis for further research. There are also other factors that should be investigated in relation to the outcome variables, such as income, critical life events, and other indicators of mental health. In order to investigate potential mediating effects, a next step would be to adopt a causal approach using path analysis or structural equation modelling.

A behavioural economic perspective was added into the interpretation of the findings to provide additional depth and theoretical context. The theory of hyperbolic discounting was not measured in the survey but applied as a conceptual framing to investigate behavioural patterns in financial and coping behaviour. Behavioural economics often relies on experimental or longitudinal methods for causal relations, thus here it is applied as a conceptual addition rather than an empirical analysis. Since it was not investigated in this particular survey, and it is not necessarily suitable for a cross-sectional study, the behavioural economic perspective should be seen as a way to enhance the interpretation of findings and to consider potential underlying processes. The inclusion of this perspective aimed to act as an initiative for further research and exploration from this perspective.

## 12. Future Research and Practical Implications

The significant correlations and predictors identified in this study call for continued exploration. Future research should focus on investigating the psychological factors underlying over-indebtedness and financial behaviour, as well as what might lead people into excessive debt. Additionally, financial literacy should be continuously investigated as a potential “protective shield” against over-indebtedness, especially together with psychological variables.

Based on our findings, several concrete and actionable suggestions can be proposed. In line with previous research and the findings of the present study, there is a clear need to include mental health assessments for individuals with excessive debt, and to promote more proactive and productive coping strategies for financial well-being. For instance, screening for financial problems in healthcare settings could help identify individuals at risk of over-indebtedness and related stress at an early stage. Furthermore, strengthening collaboration between the municipal budget and debt counselling services and primary care may further facilitate timely support for individuals experiencing financial difficulties. Reducing stigma and taboos around financial issues by framing financial health as a public health concern could also encourage individuals to seek help before their situation becomes critical. For instance, public health initiatives could promote problem-focused coping strategies, such as budgeting and seeking professional advice.

Longitudinal and interventional studies on this topic are crucial to gain a deeper knowledge about the relationship between financial perspectives and psychological variables. For instance, targeted and longitudinal interventions are recommended to identify both protective and risk factors associated with financial and psychological well-being over time. One possible approach could be to follow individuals during their final year of upper-secondary school, when they are about to enter adulthood, and continue to follow them over several years to examine how their financial and psychological health develops.

Furthermore, investigating attitudes towards consumer loans and different personal payment preferences could shed additional light on the psychological aspects of financial behaviour, particularly concerning the attitude towards credit payment stated in the report by the Swedish Consumer Agency (2023b). The amount of people in debt in Sweden is alarming, for instance amongst young individuals, and the results of this study could be of interest for policymakers seeking to reduce excessive debt, address the psychological burdens associated with early-life indebtedness, and to promote healthier financial choices. The proportion of financially literate individuals in this sample is low, at only 20 percent, further highlighting the importance of integrating financial literacy education at multiple levels of the school system to better prepare young people for the financial responsibilities of adult life, such as understanding short-term credit options like BNPL and the potential consequences of taking out consumer loans.

## 13. Conclusions

The current study has found significant associations between financial literacy, coping behaviour, and consumer financial behaviour, and how these might differ according to gender. The results indicate that a lack of financial literacy and higher levels of avoidant coping are related to the tendency to use deferred payment methods, while having financial literacy and higher levels of problem-focused coping is related to the tendency to use direct payment methods. The results suggest an increased tendency to use credit payment and consumer loans amongst men. These significant connections highlight the importance of the further exploration of psychological aspects in the financial context, and a need for increased societal financial literacy. Television shows such as ‘Squid Game’ not only provide thrilling action and exciting storylines, but also a representation of the contemporary psychological and systematic struggles of being indebted.

## Figures and Tables

**Table 1:** Demographic information of participants (*n* = 2057).

Characteristics	*n*	*%*	M (SD)	Min–Max
**Gender**				
Man	998			
Woman	1059			
**Age**			23.6	18–29
**Highest education**				
Primary	255	12.4		
Secondary	1032	50.2		
University (up to 2 years)	332	16.1		
University (more than 2 years/diploma)	438	21.3		
**Living situation**				
House	429	20.9		
Apartment	386	18.8		
Rental apartment	843	41		
Live-in or subletting	73	3.5		
Home with parents	279	13.6		
Other	47	2.3		

**Table 2:** Descriptive results for all variables by gender.

Group	*n*	% FL	% Loans	% BNPL	% Credit	% Partial	% Direct	PF M	EF M	AV M
Male	998	11.9	15.5	12.9	1.1	2.4	30.7	20.3	28.9	17.5
Female	1059	8.1	10.8	19.9	2.5	3.1	27.4	21.1	30.2	17.4
Total	2057	19.9	26.4	32.8	3.6	5.5	58	20.7	29.6	17.4

Note. FL = financially literate, Loans = have consumer loans, PF = problem-focused coping, EF = emotion-focused coping, AV = avoidant coping, Direct = use direct payment, BNPL = use the Buy-Now-Pay-Later model, Credit = use credit payment, Partial = use partial payment, and M = mean.

**Table 3:** Correlation matrix on consumer loans, financial literacy, problem-focused coping, emotion-focused coping, and avoidant coping.

Correlation Matrix		Consumer Loans	Financial Literacy	Problem- Focused	Emotion- Focused	Avoidant
Consumer loans	Pearson’s r/Phi (Φ)	-				
Financial literacy	Phi (Φ)	0.163	-			
Problem-focused	Pearson’s r df	0.141 *** 2055	0.120 *** 2055	-		
Emotion-focused	Pearson’s r df	0.033 2055	0.018 2055	0.533 *** 2055	-	
Avoidant	Pearson’s r df	−0.151 *** 2055	−0.177 *** 2055	0.046 2055	0.419 *** 2055	-

Note. Problem-focused = problem-focused coping, Emotion-focused = emotion-focused coping, Avoidant = avoidant coping, *p* < 0.001 = ***, and *p* < 0.001 (Phi).

**Table 4:** Binomial logistic regression analysis predicting consumer loan use from financial literacy, coping styles, and gender.

Predictor	Estimate	SE	Wald	OR	95% CI for OR	*p*
Intercept	1.038	0.33	9.63	2.82	[1.47, 5.44]	0.002
Financial literacy	1.05	0.17	39.72	2.87	[2.07, 3.98]	<0.001
Problem-focused coping	0.07	0.02	20.77	1.07	[1.04, 1.10]	<0.001
Emotion-focused coping	0.01	0.01	0.41	1.01	[0.99, 1.04]	0.522
Avoidant coping	−0.008	0.01	34.07	0.92	[0.89, 0.95]	<0.001
Gender (male)	−0.62	0.11	34.23	0.54	[0.44, 0.66]	<0.001

Note. *n* = 2057. The dependent variable is consumer loan (Yes v. No). OR = odds ratio; CI = confidence interval; SE = standard error; Estimate = log-odds estimate. Reference categories: Financial literacy = yes, gender = female.

**Table 5:** Significant predictors on the use of BNPL, credit, partial, and direct payment from financial literacy, coping styles, and gender.

Predictor	Payment Type	Estimate	SE	Wald	OR	95% CI for OR	*p*
Female	BNPL	0.52	0.10	27.51	1.69	[1.39, 2.05]	<0.001
Problem-focused coping	Credit	−0.08	0.04	4.97	0.94	[0.86, 0.99]	0.026
Avoidant coping	Credit	0.12	0.03	13.25	1.13	[1.06, 1.20]	<0.001
Female	Credit	−0.69	0.26	6.98	0.50	[0.30, 0.84]	0.008
Financial literacy	Partial	−1.13	0.40	7.95	0.32	[0.15, 0.71]	0.005
Problem-focused coping	Partial	−0.062	0.03	4.47	0.94	[0.89, 1.00]	0.034
Avoidant coping	Partial	0.13	0.027	24.12	1.14	[1.08, 1.21]	<0.001

Note. *n* = 2057. The dependent variable is payment preference. OR = odds ratio; CI = confidence interval; SE = standard error. Reference categories: payment type = direct payment, financial literacy = yes, and gender = male. Only statistically significant results (*p* < 0.05) are included.

## Data Availability

The data presented in this study is available on request from the corresponding author due to ethical restrictions. Although the data used in this study is anonymous, respondents were not informed in the information letter that their responses could be made publicly available. Therefore, for ethical reasons, the data is not publicly accessible.
